# Gut bacteria mediated adaptation of diamondback moth, *Plutella xylostella,* to secondary metabolites of host plants

**DOI:** 10.1128/msystems.00826-23

**Published:** 2023-11-01

**Authors:** Xiaofeng Xia, Qian Wang, Geoff M. Gurr, Liette Vasseur, Shuncai Han, Minsheng You

**Affiliations:** 1 State Key Laboratory of Ecological Pest Control for Fujian and Taiwan Crops, Institute of Applied Ecology, Fujian Agriculture and Forestry University, Fuzhou, China; 2 International Joint Research Laboratory of Ecological Pest Control, Ministry of Education, Fujian Agriculture and Forestry University, Fuzhou, China; 3 Key Laboratory of Integrated Pest Management for Fujian‐Taiwan Crops, Ministry of Agriculture and Rural Affairs, Fuzhou, China; 4 Fujian‐Taiwan Joint Innovation Centre for Ecological Control of Crop Pests, Fujian Agriculture and Forestry University, Fuzhou, China; 5 Graham Centre, Charles Sturt University, Orange, New South Wales, Australia; 6 Department of Biological Sciences, Brock University, St. Catharines, Ontario, Canada; Duke University School of Medicine, Durham, North Carolina, USA

**Keywords:** *Plutella xylostella*, secondary metabolites, gut bacteria, kaempferol, herbivorous insect, co-evolution

## Abstract

The diamondback moth (DBM), *Plutella xylostella,* has successfully adapted to the potent chemical defenses of Brassicaceae plants that deter most other herbivores. Gut bacteria are increasingly recognized as key to the biology of many species but their role in DBM adaptation to plant defense compounds is not well known. In this study, the secondary metabolites of radish seedlings, rich in flavonoids, were identified by liquid chromatography-mass spectrometry. These secondary metabolites reduced the larval growth of DBM lacking gut bacteria. The effect was rapidly eclipsed by the re-introduction of gut microbiota, which was dominated by *Enterobacter* (Proteobacteria). Similarly, while treatment with the flavonoid kaempferol adversely affected growth and extended the development time, these were alleviated by the re-introduction of *Enterobacter* sp. EbPXG5 (EbPXG5) to the DBM gut. EbPXG5 not only degrades kaempferol both *in vitro* and DBM gut, but is also shown to colonize the gut epithelium, forming a protective biofilm. Genomic sequencing of EbPXG5 showed that metabolic genes were the most abundant, especially those involved in xenobiotic degradation, and the metabolism of terpenoids and polyketides, which could participate in the degradation of plant secondary metabolites such as kaempferol. Overall, our results showed that EbPXG5 is a bacterium common in the gut of DBM larvae and has the *in vitro* and *in vivo* capacity to detoxify a major secondary metabolite that is produced in brassica plants as a defense against herbivores. This insect-bacterial association may be an important contributor to the status of DBM as a major pest of brassica crops worldwide.

**IMPORTANCE:**

In this study, we identify an important role of gut bacteria in mediating the adaptation of diamondback moth (DBM) to plant secondary metabolites. We demonstrate that kaempferol’s presence in radish seedlings greatly reduces the fitness of DBM with depleted gut biota. Reinstatement of gut biota, particularly Enterobacter sp. EbPXG5, improved insect performance by degrading kaempferol. This bacterium was common in the larval gut of DBM, lining the epithelium as a protective film. Our work highlights the role of symbiotic bacteria in insect herbivore adaptation to plant defenses and provides a practical and mechanistic framework for developing a more comprehensive understanding of insect-gut microbe-host plant co-evolution.

## INTRODUCTION

Plant secondary metabolites refer to a large group of end products produced by a complex suite of metabolic pathways in plants. Plant secondary metabolites do not directly participate in growth, development, and reproduction, but play an important role in many aspects of plant biology, such as adaptation to environmental stress and resistance to pathogens and herbivores (1, 2). Plant secondary metabolites mainly include nitrogen compounds, terpenoids, flavonoids, and phenolic compounds, which vary in chemical structures and among species (2). Plant secondary metabolites, such as nitrogen and sulfur compounds (a kind of plant glycosides with nitrogen or sulfur), can improve the ability of plants to resist the adverse effects of predators, competitors, and parasites. For example, mustard glycosides in cruciferous plants can play a toxic role to DBM (2, 3). Phenolic compounds, such as chlorogenic acid, have a direct toxic effect on insects (4, 5). Terpenoids, such as monoterpene pyrethroids, enhance plant defense against phytophagous insects. These compounds are widely used as commercial insecticides and have activity against beetles, wasps, moths, and bees (6–8). A volatile homoterpene compound—(3E)-4,8-dimethyl-1,3,7-nonatriene—is toxic to DBM and destroys its peritrophic membrane, resulting in midgut microbial-mediated insecticidal effect (9). Plant secondary metabolites can be exploited by specialist herbivores for the detection and selection of host plants (10, 11), or even sequestered to provide the herbivore with defenses against its natural enemies (12). Ultimately, however, the fitness of the herbivore demands that it can metabolically overcome these plant secondary metabolites, potentially by detoxification.

DBM is a worldwide pest with a wide resistance to insecticides, threatening cruciferous crop production (13, 14). DBM mainly feeds on cruciferous plants, which contain glucosinolates, sulfur-containing secondary metabolites (3), as well as flavonoids (quercetin, kaempferol, isorhamnetin, etc.) (15, 16). In the long-term co-evolution of plants and insects, a set of defense and counter-adaptation systems have developed. Accordingly, plant secondary metabolites are a major driving force in the co-evolution of plants and insects (17). In this “arms race,” both sides strive to make themselves better adapted and maximize fitness (18–20).

In insects, the gut is responsible for food digestion and nutrient absorption and is now known to be inhabited by a strikingly large number and diversity of microorganisms. These microbes form symbiotic relationships for mutual benefit (21, 22), contributing to the host’s nutrition, absorption, metabolism and detoxification, development and reproduction, resistance to pathogens, immunity, and pesticide resistance (23, 24). The gut bacteria of insects have co-evolved with their host to enable the degradation of plant secondary metabolites and help insects adapt to host plants. Gut bacteria of coffee berry borer (*Hypothenemus hampei*), for instance, contribute to detoxifying the caffeine, which is toxic to other herbivores (25). The gut bacteria in *Bemisia tabaci* differ when reared on different plants; insects reared on pepper had microbiomes enriched with *Mycobacterium* and had an abundance of degradative pathways of xenobiotics and secondary metabolites (26). Our previous study based on metagenomics analysis shows that the DBM gut microbiome contains a conserved pathway for phenol degradation, which may play a role in the detoxification of the plant secondary metabolites (27).

Previous studies have found that the gut microorganisms of DBM are mainly composed of Proteobacteria and Firmicute, and the proportion of *Enterobacter* in the gut symbiosis is over 70% (27–29). A great difference in microorganisms in the gut of DBM can be found between chlorpyrifos-resistant and susceptible DBM strains. The abundance of Firmicute in resistant strains is significantly higher than that in sensitive strains, and this abundance can be enhanced with increased pesticide stress (29). A functional study suggests that *Enterococcus*, a Firmicute, can mediate the resistance of DBM to chlorpyrifos (30). The gut bacteria of DBM can affect the fitness of the host, its development duration, and adult life span, as well as larval and pupal weights (31). DBM gut microorganisms significantly contribute to the degradation of lignocellulose and detoxification of phenols, suggesting that the gut microorganism of DBM may assist the host to adapt to the plant through food digestion and secondary metabolites detoxification (27).

Some biological functions of gut microorganisms of DBM have been described. The exploration of the role and the mechanism of gut microbes in the resistance of DBM to plant secondary metabolites, however, has just begun. Understanding the mechanism by which DBM responds to plant secondary metabolites’ stress not only provides ideas for potential new pest control tactics but is also a good model for revealing more fundamental aspects of microbiome-mediated co-evolution among plants and insects. In the present study, we aimed to clarify and discuss the following questions: (i) Do plant secondary metabolites of radish adversely affect DBM? (ii) What is the role and mechanism of gut microorganisms in the response of DBM to the relevant plant secondary metabolites? Since the DBM only feeds on plants at the larval stage, the research focused on plant secondary metabolites at the larval stage and studied their relationship by establishing a model of “DBM larvae–gut microbiota–plant secondary metabolites.”

## MATERIALS AND METHODS

### Extraction of secondary metabolites from radish seedlings

Leaves of radish seedlings were cut, washed, vacuum dried, and mixed with ethanol (Sinopharm Chemical Reagent Co., Ltd, China) in a weight ratio of 8:1. The mixture was placed in a water bath to evaporate the solvent at a constant temperature of 70°C, which was collected in an evaporator flask. This process was repeated twice, and the two solutions were mixed. The solvent of the solutions was evaporated using a rotating evaporator, and once in a paste, the extract was mixed with a small amount of methanol (Sinopharm Chemical Reagent Co., Ltd, China). This mixture was then ultrasonically dissolved, transferred to a small beaker, and dried under vacuum to constant weight. The extracted secondary metabolites were stored at 4°C in a sealed and dark container for further study.

### Production of bacteria-free DBM larvae

The methods for the production of sterile DBM larvae are detailed in our earlier publication (32). Briefly, eggs of DBM fed on an artificial diet (hereafter termed SLss) were collected and disinfected. They were washed with sterile water for 3 min, a 1.5% NaClO solution for 30 s, 75% alcohol for 3 min, and sterile water twice for 3 min each time, then the eggs were dried. After hatching, the larvae were fed on the same artificial diet, with all materials used being disinfected or sterilized. The artificial diet was sterilized as follows: part A was 37.5 g wheat germ powder, 20.0 g yeast powder, 10.0 g sucrose, 3.0 g radish seed, 1 mL rapeseed oil, 30 µL linoleic acid, and 6.0 g agar, mixed and added into 250 mL deionized water, stirred, and sterilized at 115°C for 30 min under high pressure. Part B included 0.5 g Nipagin, 1.0 g sorbic acid, 0.8 g complex vitamin, and 1.0 g vitamin C dissolved in about 5 mL deionized water, filtered, and sterilized. Part B was added to the sterilized part A to get the germ-free artificial diet. The larvae were fed with this artificial diet. Adults were fed with 10% honey. The rearing conditions were temperature 25°C ± 2°C, humidity 70%– 80%, light cycle 16L:8D.

To verify that the obtained insects were the DBM without gut bacteria, the DBM reared under the sterilized artificial diet up to the fourth instar were collected and dissected to collect the gut contents under sterile conditions. Luria-Bertani (LB) plates were used to detect bacterial clones from the gut contents. The total DNA of the gut contents was used as a template to amplify the bacteria 16S rDNA to eliminate the possible presence of non-culturable bacteria. DBM reared on plants under normal conditions were used as control.

### Fitness effect of secondary metabolites of radish seedlings on DBM

Artificial diets containing radish seedling extract solutions with the final concentration of 1 or 0.1 mg/mL were prepared, and the solvent methanol was used for control (CK). After hatching, the first instar larvae of DBM were collected, and 30 larvae were randomly selected to feed on the artificial diets. When the larvae grew to the fourth instar, each was weighed, and the average weight of the fourth instar larvae was calculated. Larvae were then allowed to develop to pupation, and the development time was recorded. This experiment was run three times. Data were analyzed by independent one-way ANOVA in IBM SPSS (Statistical Product and Service Solutions) 19 software.

### Effect of gut bacteria on the adaptation of DBM larvae to plant secondary metabolites

Ten fourth instar larvae of DBM feed on radish seedlings (hereafter termed FZss) were randomly selected. After body surface disinfection, they were dissected and the gut contents were homogenized in a sterile microcentrifuge tube (EP) tube with 1 mL sterile water. One hundred microliter of gut content homogenate was removed and inoculated into LB liquid medium, cultured under 37°C, and shaken overnight. The bacterial culture solution was then centrifugated for 10 min at 6,000 rpm. The precipitate was washed with sterile water three times and diluted with 1 mL sterilized water to get the mother liquor of simulated gut bacteria (DBMT). Fifty first instar larvae of DBM were randomly collected to feed on the artificial diet that contained the radish seedling extract with the final concentration of 0.1 mg/mL (CK + R), or both with the radish seedling extract (0.1 mg/mL) and the DBMT (OD_600_ = 0.1) (CK + R + B), the control group had a corresponding volume of sterile water. The average weight of the fourth instar larvae and the larval development time were recorded. Data were analyzed by one-way ANOVA in IBM SPSS 19 software.

The microbial diversity of the DBMT was analyzed by denaturing gradient gel electrophoresis (DGGE) to verify whether the main components of the LB-enriched gut microbes (DBMT samples) are consistent with the normal gut microbes of the DBM, so as to evaluate the feasibility of using the LB-enriched gut microbes’ method to build a simulated gut microbiome. The total DNA of DBMT was extracted by QIAamp DNA Stool Mini Kit (Qiagen, Germany). Taking the genomic DNA as template, the bacterial 16S rDNA universal primers 27F and 1492R (27F: 5′-AGAGTTTGATCCTGGCTCAG-3′, 1492R: 5′- GGTTACCTTGTTACGACTT-3′) were used for PCR amplification. PCR reaction system was: 12.5 µL 2× Phanta Max Buffer, 0.5 µL dNTP Mix, 0.5 µL Phanta Max Super-Fidelity DNA Polymerase, 1 µL 27F (2 µM), 1 µL 1492R (2 µM), 1 µL gDNA (1 ng /µL), and 8.5 µL nuclease-free water. Amplification conditions were as follows: 94°C for 3 min, 94°C for 30 s, 55°C for 30 s, 72°C for 1 min, 30 cycles, and 72°C for 10 min. The PCR product was detected by 1.0% agarose gel electrophoresis, and then the PCR product was used as the second template. The 16S rDNA V3 universal primers 343F + GC and 534R (343F + GC: 5′-CGCCCGCCGCGCGCGGCGGGCGGGGCGGGGGC-3′, 534R: 5′- ATTACCGCGGCTGCTGG-3′) (33) were used for PCR amplification. The reaction system was: 12.5 µL 2× Phanta Max Buffer, 0.5 µL dNTP Mix, 0.5 µL Phanta Max Super-Fidelity DNA Polymerase, 1 µL 343F + GC (2 µM), 1 µL 534R (2 µM), 1 µL gDNA (1 ng/µL), and 8.5 µL nuclease-free water. The procedure was: 94°C for 5 min, 94°C for 1 min, 65°C–55°C for 1 min, and each cycle temperature was reduced by 0.5°C for 20 cycles, 72°C for 1 min, then 94°C for 1 min, 50°C for 1 min, 72°C for 1 min with 10 cycles, and 72°C for 10 min. The PCR product was detected on 1.5% agarose gel electrophoresis, and the V3 variable region of the 16S rDNA gene was purified and recovered using the agarose gel recovery kit (Omega, USA). The concentration of denatured gel used for DGGE analysis was between 25% and 55%. The conditions of DGGE analysis were 60°C and 80 V for 13 h. After electrophoresis, the gel was fixed and stained, then photographed by Epson Gel imager as in a previous study (34). The separated bands were cut off and recovered, inserted into the plasmid pESI-Blunt simple vector by the connection kit (Yeasen, Shanghai), the vectors were then transformed to *Escherichia coli* DH5α, and the positive clones were sequenced by Biotech Boshan Co., Ltd., Shanghai, aligned with the NCBI GenBank database to identify the bacterial species.

### Analysis of secondary metabolites in radish seedlings by LC-MS

The radish seedling extract was filtered with a 0.45 µm microporous filter membrane and transferred into a liquid phase vial for detection. A radish seedling extract of 1 µL was injected into the instrument by an automatic sampler and analyzed by liquid chromatography-mass spectrometry (LC-MS). The conditions of the liquid phase were as follows: acetonitrile (Sigma, Germany) containing 0.1% formic acid (FA) (Thermo Fisher, USA) is mobile phase A, water containing 0.1% FA is mobile phase B, the flow rate was 0.3 mL/min, column temperature was 40°C, and sample temperature was 10°C. The elution procedure was as follows: 0–5 min, 0%–5% A; 5–7.5 min, 5%–15% A; 7.5–30 min, 15%–85% A; 30–36 min, 85%–100% A; 36–52 min, 100% A; 52–53 min, 100%–0% A; 53–56 min, 0% A. The temperature of the capillary was 450°C, the capillary voltage was 2.5 kV in the negative ion mode and 3 kV in the positive ion mode, respectively. The scanning range of mass spectrometry was *m*/*z*: 50–1,000.

### Fitness effect of kaempferol on DBM

Kaempferol is commonly found in high abundance in cruciferous vegetables and can play an important protective role in plants against *Spodoptera litura* attack (35–37). In this study, it was selected to further identify its interaction with DBM and gut bacteria. Kaempferol (Solarbio, China) was solubilized in methanol and added to the germ-free artificial diet to make the final concentration 0.1 mg/mL. Thirty first instar DBM larvae were randomly selected to feed on this artificial diet, and another 30 were used to feed on the artificial diet with the same volume of solvent as CK. The fourth instar larval weight and the larval development time were recorded, and the experiment was run three times. Data were analyzed using a one-way ANOVA in IBM SPSS 19 software.

### Effect of kaempferol on gut bacterial diversity of DBM

Eggs of DBM were collected and disinfected (as described above) and, when hatched, 20 first instar larvae were randomly selected and reared under the germ-free artificial diet to second instar. These were transferred to the artificial diet containing DBMT (OD_600_ = 0.1) described above for 24 h. Then, the larvae were transferred to an artificial diet containing kaempferol (0.1 mg/mL) and reared up to the fourth instar (DBMT-KAE); the control group was fed a sterile diet without kaempferol (DBMT-CK). This experiment was run three times. Fourth instar larvae reared under the above diets were dissected under sterile conditions, and the gut contents were collected. The metagenomic DNA of the gut microbiota of the samples was extracted by QIAamp DNA Stool Mini Kit (Qiagen, USA). DNA samples were diluted with sterile water to a final concentration of 1 ng/µL, and then sent to Novogene Co. Ltd. for subsequent sequencing (V3–V4 region of bacterial 16S rDNA gene was sequenced using Illumina HiSeq 2500 platform) and analysis based on the company’s production process. Cutadapt (V1.9.1, http://cutadapt.readthedocs.io/en/stable/) was first used to filter out the low-quality of the reads, and then the barcode and primer were cutoff to get the raw data (raw reads) (38). The raw reads were compared with the species annotation database (https://github.com/torognes/vsearch/) to remove the chimeric sequences to get the effective data (clean reads) (39). Uparse software (Uparse v7.0.1001, http://www.drive5.com/uparse/) was used to cluster clean reads, and the sequences with 97% identity were clustered into operational taxonomic units (OTUs) (40, 41). The representative sequences of OTUs were selected to annotate the species. The species annotation analysis was carried out by using the Mothur and the SSU rRNA database of silva132 (http://www.arb-silva.de/) to get the relevant taxonomic information. The community composition of each sample was counted at the taxonomic level (42). The unifrac distance was calculated, and the UPGMA tree was constructed by QIIME software (version 1.9.1).

### Effect of gut bacteria EbPXG5 on the adaptability and survival of DBM to kaempferol

The *Enterobacter* sp. EbPXG5 (GenBank accession number: JQ396388) was isolated from the DBM gut, and 16S rDNA sequence alignment and genome analysis both indicate that the bacterium was classified as *Enterobacter cloacae*. The strain is currently stored in the State Key Laboratory of Ecological Pest Control for Fujian and Taiwan Crops, Fujian Agriculture and Forestry University. The bacterium was inoculated into an LB liquid medium and cultured at 37°C overnight, and then the culture solution was centrifuged for 10 min at 6,000 rpm. The precipitate was washed with sterilized water three times and dissolved with an appropriate amount of sterilized water. Thirty first instar larvae of DBM were randomly collected to feed on the artificial diet containing the kaempferol with the final concentration of 0.1 mg/mL or both the kaempferol (0.1 mg/mL) and the EbPXG5 (OD_600_ = 0.1). The control group received the same volume of methanol and sterilized water. The fourth instar larval weight and the larval development time of DBM were observed based on the above method. This experiment was repeated three times. Data were analyzed by one-way ANOVA in SPSS 19 software.

### Analysis of degradation of kaempferol by EbPXG5 *in vitro*

The EbPXG5 dissolved in the sterile water (OD_600_ = 1.0) was inoculated into the minimal salt medium, MSM (1.0 g K_2_HPO_4_, 0.3 g KH_2_PO_4_, 0.1 g MgSO_4_·7H_2_O, 1.0 g NaCl, 1.0 g NH_4_NO_3_, dissolved in 1 L sterile water, mixed well, and pH adjusted to 7.0, then autoclaved at 121°C for 20 min) at an inoculation amount of 10% (vol/vol). Kaempferol was added to the MSM medium to reach the final concentration of 2 mg/mL. The bacteria were cultured using a shaker at 30°C, 180 rpm for 48 h, then centrifuged at 12,000 rpm for 5 min. The supernatant was transferred to a new tube and dried on a nitrogen-blowing instrument. The sediment was dissolved in 200 µL of 70% methanol and filtered into the liquid phase vial with 0.45 µm filter membrane. LC-MS was used to detect the change of kaempferol degraded by EbPXG5. The experiment was repeated three times as well as in the control medium without EbPXG5. The data of chromatographic peak area were log10 transformed and then analyzed by independent sample *t*-test in SPSS 19 software. To detect the growth curve of EbPXG5, 1.0 mL culture medium was taken out to detect the OD_600_ value every 12 h (0, 12, 24, 36, and 48 h). An extraction solution of 1 µL stored in the liquid phase vial was injected into the LC-MS by auto-sampler for analysis. The liquid phase conditions were as follows: acetonitrile containing 0.1% FA is mobile phase A, water containing 0.1% FA is mobile phase B, the flow rate is 0.3 mL/min, the column temperature is 40°C, and the sample temperature is 10°C. According to the following gradient elution: 0–2 min 0%–2% A, 2–14 min 2%–30% A, 14–16 min 30%–50% A, 16–18 min 50%–70% A, 18–20 min 70%–98% A, 20–23 min 98% A, and 23–26 min 98%–0% A. The capillary temperature was 450°C, the capillary voltage was 2.5 kV in the negative ion mode, and the scanning range of the mass spectrum was *m*/*z*: 50–1,000.

### Degradation of kaempferol by EbPXG5 *in vivo*

The eggs of DBM were collected and surface disinfected and, when eggs hatched, 50 first instar larvae were randomly selected and reared on either the germ-free artificial diet containing kaempferol (0.1 mg/mL) or a diet with the kaempferol (0.1 mg/mL) and EbPXG5 (OD_600_ = 0.1) to grow to the fourth instar. Six sets of 50 larvae per treatment were used. The feces of the fourth instar larvae were collected, and 0.05 g of feces was transferred into a 1.5 mL centrifuge tube. To this, 400 µL of 70% methanol solution was added for kaempferol extraction. The tubes were then shaken on the vortex shaker for 1 min, and then crushed by an ultrasonic crusher for 20 min. After crushing, the samples were centrifuged at 12,000 rpm for 1 min at 4°C, the supernatant was transferred to new tubes and centrifuged again at 12,000 rpm for 20 min at 4°C. The supernatant was then transferred and diluted to 1:10 times, filtered by 0.45 µm filter membrane, and transferred into a liquid phase vial for detection under LC-MS (the analysis method as described earlier).

### Colonization of EbPXG5 in the gut of DBM

#### Fluorescence *in situ* hybridization (FISH) analysis of the DBM gut of the SLss strain

The eggs of SLss were collected for surface disinfection and, when eggs hatched, neonates were reared under the germ-free artificial diet up to second instar. These were transferred to the artificial diet containing EbPXG5 (OD_600_ = 0.1) to grow to the fourth instar and then starved for 12 h. These DBM were dissected in a sterile environment. The dissected guts were soaked in 4% paraformaldehyde tissue fixative solution and fixed overnight at 4°C. The guts were embedded with optimum cutting temperature compound (Sakura, Japan) and cut into 10 µm thickness in a freezing microtome. The thin sections were collected and placed on pre-cooled slides. The embedding agent around the sections was slowly washed with anhydrous ethanol, and the slices were cleaned with PBS buffer for three times, each time for 5 min. The slides were then hybridized with 1 µM of each probe in the preheated (50°C) hybridization solution (0.02 M Tris-HCl [pH 8.0], 3.3% formamide, 0.1 M NaCl, 1% SDS). The online primer design software (http://www.oligoarchitect.com/oligoarchitect/loginservlet) was used to design the probe primer of EbPXG5 (*Enterobacter* sp. Probe AA: Cy5-CACACTGGAACTGAGACACGGT) according to the 16S rDNA sequence, and the probe for Enterobacteriaceae (Probe DD: Hex-TGCTCTCGCGAGGTCGCTTCTCTT) was synthesized according to the previous study (43, 44). All of the probes were synthesized by BioSune Bioengineering Co., Ltd (Shanghai, China). The hybridization was conducted at 50°C for 5 h in darkness; after that, the slides were eluted with 20 µL of preheated eluent (1% SDS, 3 mM NaCl, and 2 mM sodium citrate) for 10 min in darkness. The sections were finally washed with PBS five times, 5 min each time. When the slices were slightly dry, 4',6-diamidino-2-phenylindole (DAPI) was added, and coverslips were put in place and sealed with nail polish. Guts without EbPXG5 were used as the control. SP8 laser scanning confocal microscope (Leica, Germany) was used to observe and take photos.

#### FISH analysis of the DBM gut of the FZss strain

To analyze the distribution of EbPXG5 or other Enterobacteriaceae in the gut of DBM feeding on plants, fourth instar larvae of FZss reared on the radish seedlings were collected and starved either for 0 or 12 h. Their guts were dissected, sectioned, hybridized, and observed with the same methods as described for SLss, except that DAPI was not used to stain the nuclei.

### Sequencing and analysis of the whole genome of EbPXG5

The EbPXG5 was collected and sent to Beijing Genomics Institute (BGI, Shenzhen, China) to extract genomic DNA and perform sequencing. The EbPXG5 genome was sequenced using a PacBio RS II platform and an Illumina HiSeq 4000 platform. The assembly of the genome was divided into three parts: (i) subreads produced by PacBio were corrected by the programs Pbdagcon and FalconConsensus, and then subreads of <1 kb were removed to get the high-quality CorrectedReads. (ii) Celera (version 8.3, http://sourceforge.net/projects/wgs-assembler/files/wgs-assembler/wgs-8.3/) and Falcon (version v0.3.0, https://github.com/PacificBiosciences/falcon) were used to assemble the CorrectedReads, then the best assembly result was chosen. (iii) Illumina Hiseq data were used for single base error correction, and the GATK (version 1.6–13, http://www.broadinstitute.org/gatk/) and SOAPsnp/SOAPindel (45) were used to get the highly reliable assembly genome. By comparing the genome with the Nt database of NCBI, EbPXG5 was identified as *E. cloacae*. Gene prediction was performed on the EbPXG5 genome assembly by Glimmer3 (http://www.cbcb.umd.edu/software/glimmer/) with Hidden Markov models (46). The best hit was used for function annotation by the Blast alignment tool. The databases of KEGG (Kyoto Encyclopedia of Genes and Genomes) and COG (Clusters of Orthologous Groups) were used for general functional annotation.

## RESULTS

### Effect of secondary metabolites from radish seedling on DBM

**Fig 1:**
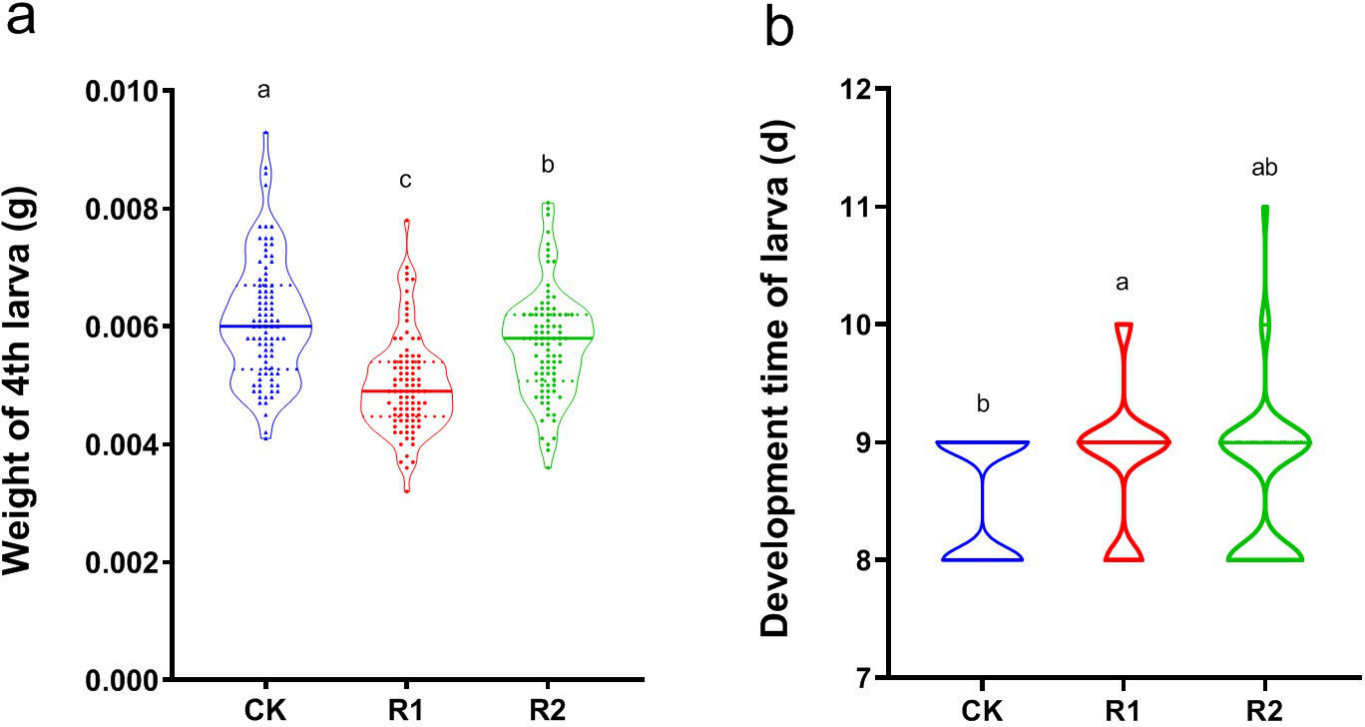
Impact of radish extract addition to the diet on the growth and development of *Plutella xylostella*. (a) Fourth instar larval weight; (b) larval development time. CK, control with standard artificial diet; R1, artificial diet with radish seedling extract, 1 mg/mL; and R2, artificial diet with radish seedling extract, 0.1 mg/mL.

After feeding on sterile artificial diet, no bacteria were detected in SLss gut both through plates’ culture and 16S rDNA amplification (Fig. S1a and b). However, bacteria were detected in the FZss gut contents (Fig. S1c), suggesting that we had obtained DBM individuals without gut bacteria, which could be used for further study. The weight of fourth instar larvae of DBM without gut bacteria reared on radish seedling extract was significantly reduced (*F*_2, 267_ = 32.388, *P* ≤ 0.001, Fig. 1a). The higher the concentration of extract, the greater the weight reduction. Radish seedling extract also prolonged larval development of DBM (*F*_2, 267_ = 6.348, *P* = 0.002, Fig. 1b).

### Effect of gut bacteria on the adaptation of DBM to the extracts of radish seedlings

**Fig 2:**
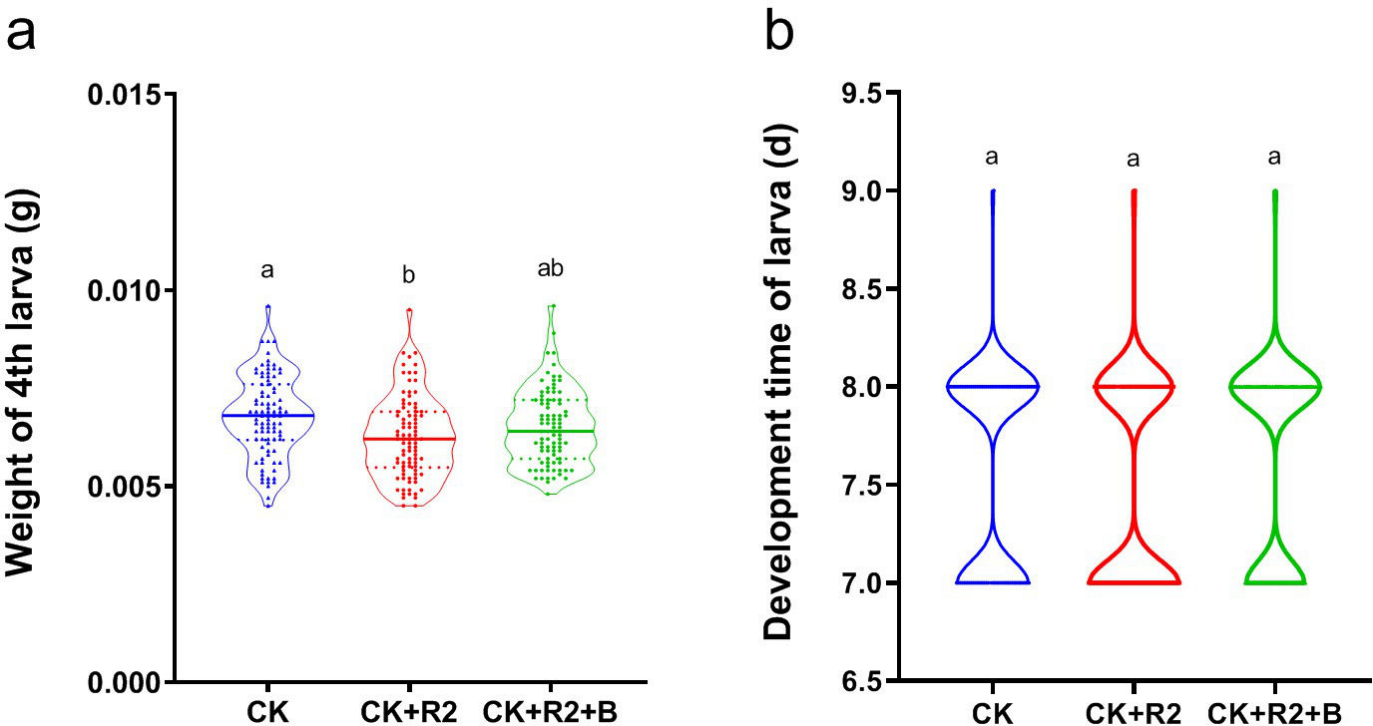
Impact of gut bacteria assisting the adaptation of *Plutella xylostella* to the host plant secondary metabolites. (a) The impact of plant secondary metabolites and the gut microbiome on the fourth instar larval weight. (b) The impact of plant secondary metabolites and gut microbiome on larval development time. CK, control with the standard artificial diet; R2, artificial diet with radish seedling extract, 0.1 mg/mL; and B, gut microbiome (OD_600_ = 0.1) enriched by LB medium using the gut contents of *P. xylostella* (DBMT).

PCR-DGGE analysis revealed that the LB-enriched gut microbes (DBMT) recovered from the DBM were composed mainly of *Pseudomonas*, *Bacillus*, *Enterobacter*, *Klebsiella*, *Erythrobacter*, *Escherichia*, and *Carnobacterium* (Fig. S2), all from the phyla Proteobacteria and Firmicutes (Fig. S2b and c), as observed in our previous study based on metagenomics sequencing (27). The addition of radish seedling extract led to a decrease in DBM weight, but the weight increased after adding bacteria to the artificial diet (*F*_2, 267_ = 5.988, *P* = 0.003, Fig. 2a). The introduction of gut bacteria had no effect on the larval stage (*F*_2, 267_ = 1.838, *P* = 0.161, Fig. 2b). *Enterobacter* was the most abundant genus in the gut (Fig. S2b and c), and it might play an important role that mediated the adaptation of DBM to the radish plant.

### Kaempferol identification in radish seedling and the effect on DBM gut bacteria

**Fig 3:**
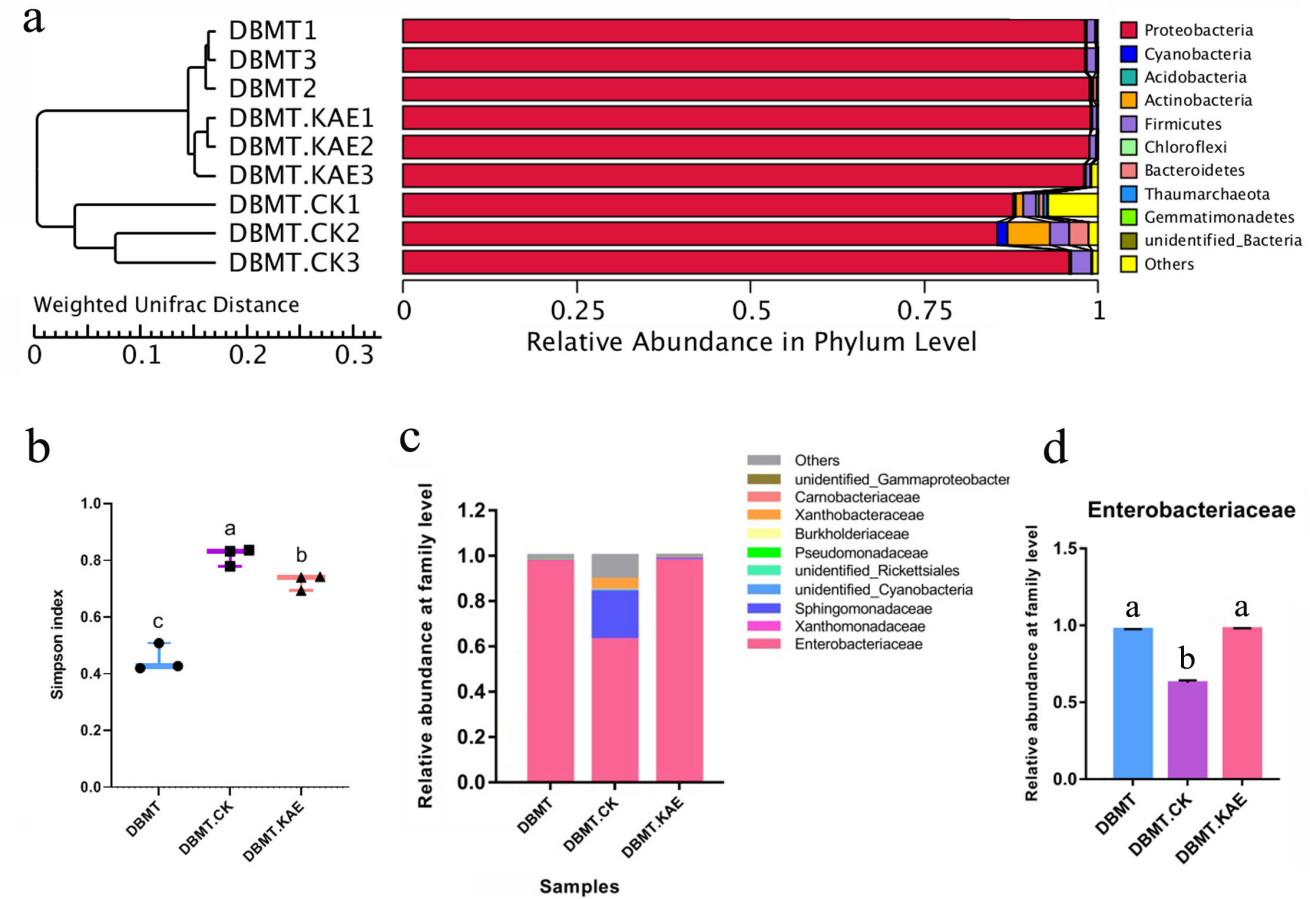
Effect of kaempferol on the gut microbial diversity of *Plutella xylostella*. (a) UPGMA clustering tree based on weighted UniFrac distance. (b) Alpha diversity of *P. xylostella* gut bacteria (Simpson index), (c) Composition of bacteria at the family level. (d) Abundance of Enterobacteriaceae among different samples. DBMT, the mixed gut microbiome enriched by LB medium using the gut contents of *P. xylostella*; DBMT.CK, second instar *P. xylostella* larvae were reared with an artificial diet containing the DBMT (OD_600_ = 0.1) for 24 h, then transferred to the sterile artificial diet to grow up to the fourth instar (DBMT.CK); DBMT.KAE, the second *P. xylostella* instar larvae reared with an artificial diet containing the DBMT (OD_600_ = 0.1) for 24 h, then transferred to the sterile artificial diet containing kaempferol (0.1 mg/mL) and grown to the fourth instar (DBMT.KAE).

Over 60 types of secondary metabolites were identified from LC-MS in the radish seedlings, including alkene, alcohol, acid, phenol, and ester. The abundance of flavonoids (kaempferol, quercetin, and isorhamnetin) was relatively high (Table S1). Gut bacterial diversity was affected by the kaempferol (Fig. 3). When kaempferol was added to the medium, the gut microbial diversity decreased (Fig. 3a and b), and the abundance of Enterobacteriaceae (Proteobacteria) increased (Fig. 3c and d). This result further supported the role of Enterobacteriaceae in the response of DBM to the plant secondary metabolites, especially the flavonoids such as kaempferol. Interestingly, the bacterial population established in the gut of DBM after feeding (DBMT.CK) greatly varied compared with DBMT (Fig. 3).

### Effect of EbPXG5 on the adaptation of DBM to kaempferol

**Fig 4:**
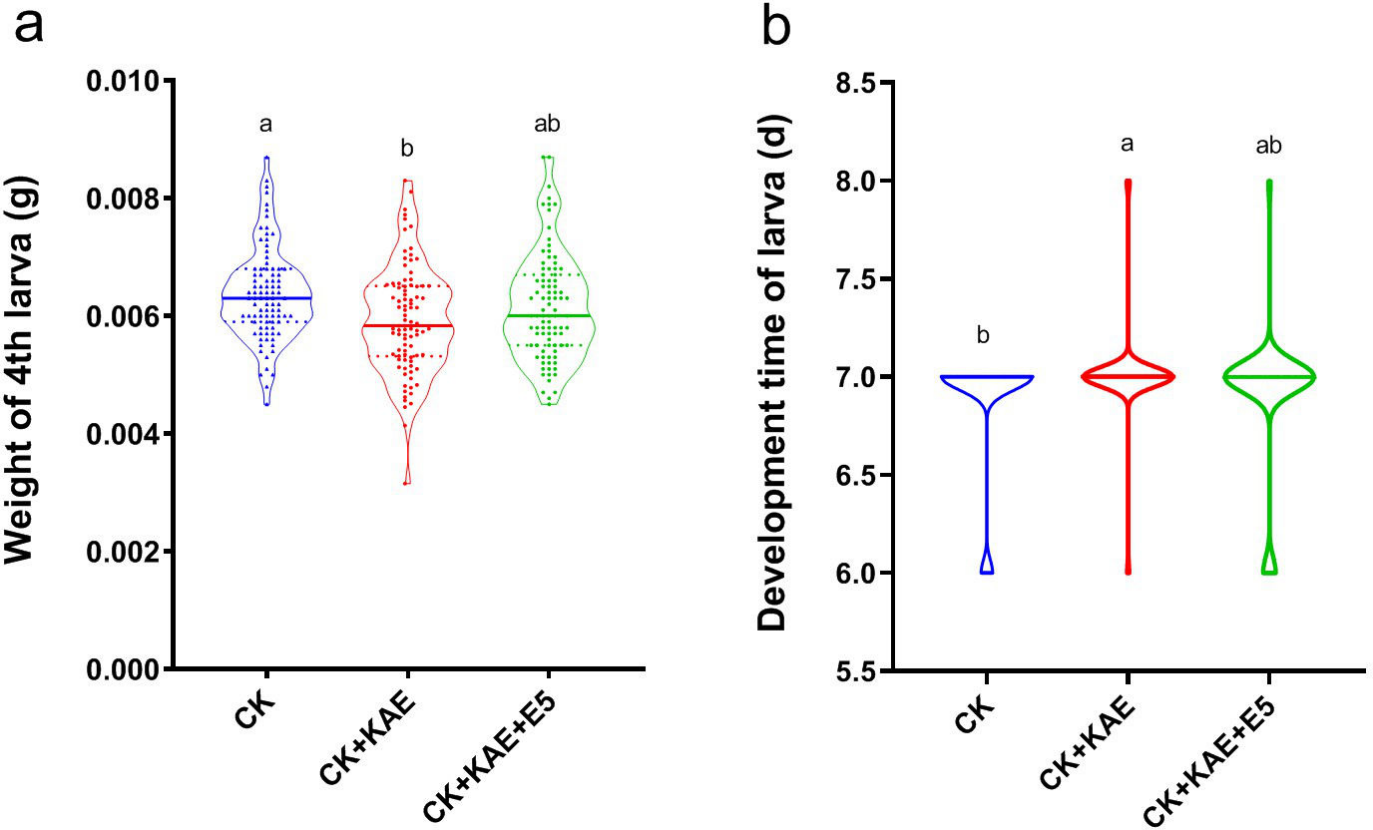
Impact of EbPXG5 assisting the adaptation of *Plutella xylostella* to the kaempferol on fitness. (a) Impact of kaempferol and EbPXG5 on the fourth instar weight. (b) Impact of kaempferol and EbPXG5 on larval development time. KAE, kaempferol, 0.1 mg/mL; E5, *Enterobacter* sp. EbPXG5 (EbPXG5), OD_600_ = 0.1.

As EbPXG5 was identified as *E. cloacae*, which was the most abundant bacterium in DBM larvae gut, it was selected for further interaction analysis. When challenged with kaempferol, the weight of the fourth instar larvae of DBM decreased, but this trend was reversed by the presence of EbPXG5 (*F*_2, 267_ = 5.580, *P* = 0.004, Fig. 4a). Similarly, kaempferol prolonged larval development time but the introduction of EbPXG5 alleviated this effect (*F*_2, 267_ = 4.225, *P* = 0.016, Fig. 4b).

### Mechanism of EbPXG5 on the adaptation of DBM to kaempferol

**Fig 5:**
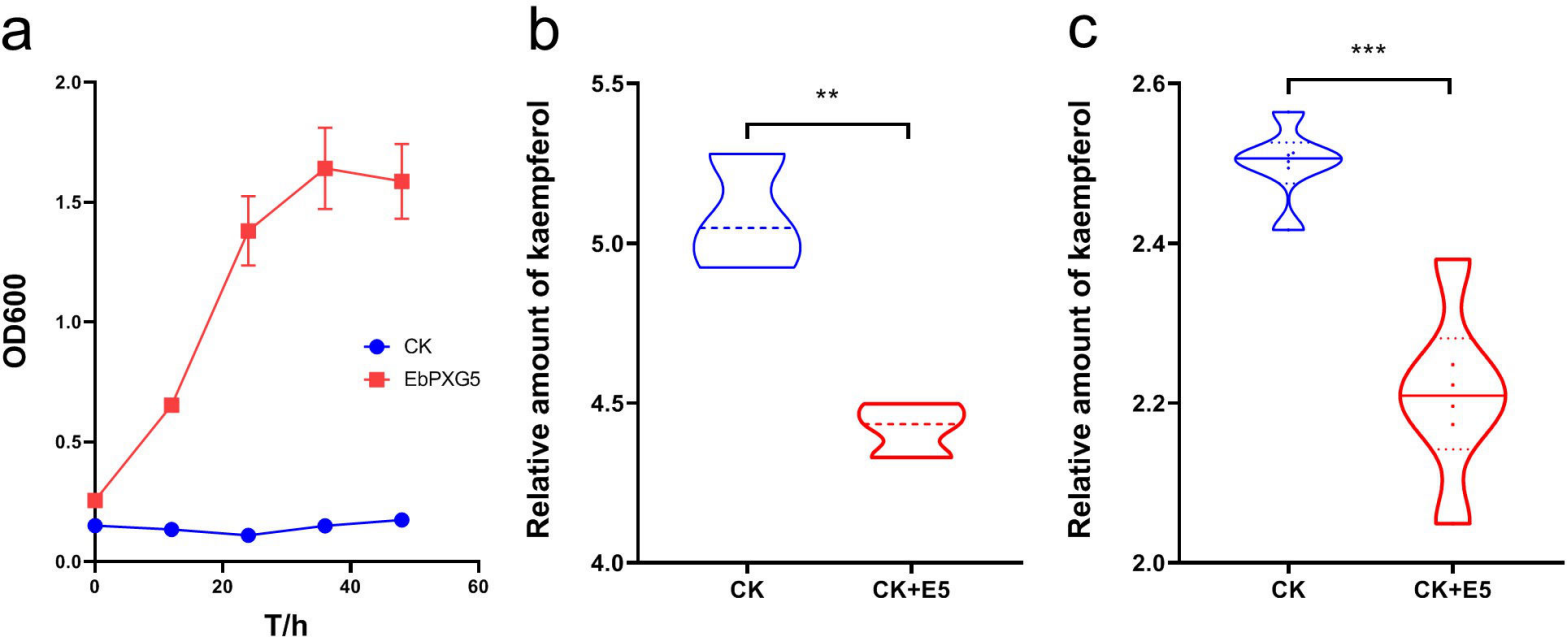
Degradation of kaempferol by EbPXG5. (a) Growth curve of EbPXG5. (b) Degradation of kaempferol by EbPXG5 *in vitro.* (c) Degradation of kaempferol by EbPXG5 in *P. xylostella* gut. Panel b: CK, the MSM containing kaempferol (2 mg/mL) and CK + E5, the EbPXG5 cultured under MSM containing kaempferol (2 mg/mL). Panel c: CK, *P. xylostella* reared with artificial diet containing kaempferol (0.1 mg/mL) and CK + E5, the *P. xylostella* reared with artificial diet containing both kaempferol (0.1 mg/mL) and EbPXG5. The data in the ordinate of both panels b and c are the data obtained after normalization of the chromatographic peak area with log10. **P < 0.01 and ***P < 0.001.

To determine whether EbPXG5 can utilize kaempferol, it was cultured in inorganic salt medium (MSM) with kaempferol as the sole carbon source. The results showed that EbPXG5 can utilize kaempferol for growth (Fig. 5a). LC-MS analysis showed that the kaempferol content in the culture medium with EbPXG5 was significantly lower than in the control group without EbPXG5 (*T* = 5.747, *df* = 4, *P* = 0.005) (Fig. 5b). Whilst those results indicated that EbPXG5 could degrade kaempferol *in vitro*, we also found that EbPXG5 could degrade kaempferol in the DBM gut environment (*T* = 6.002, *df* = 10, *P* = 0.000) (Fig. 5c).

**Fig 6:**
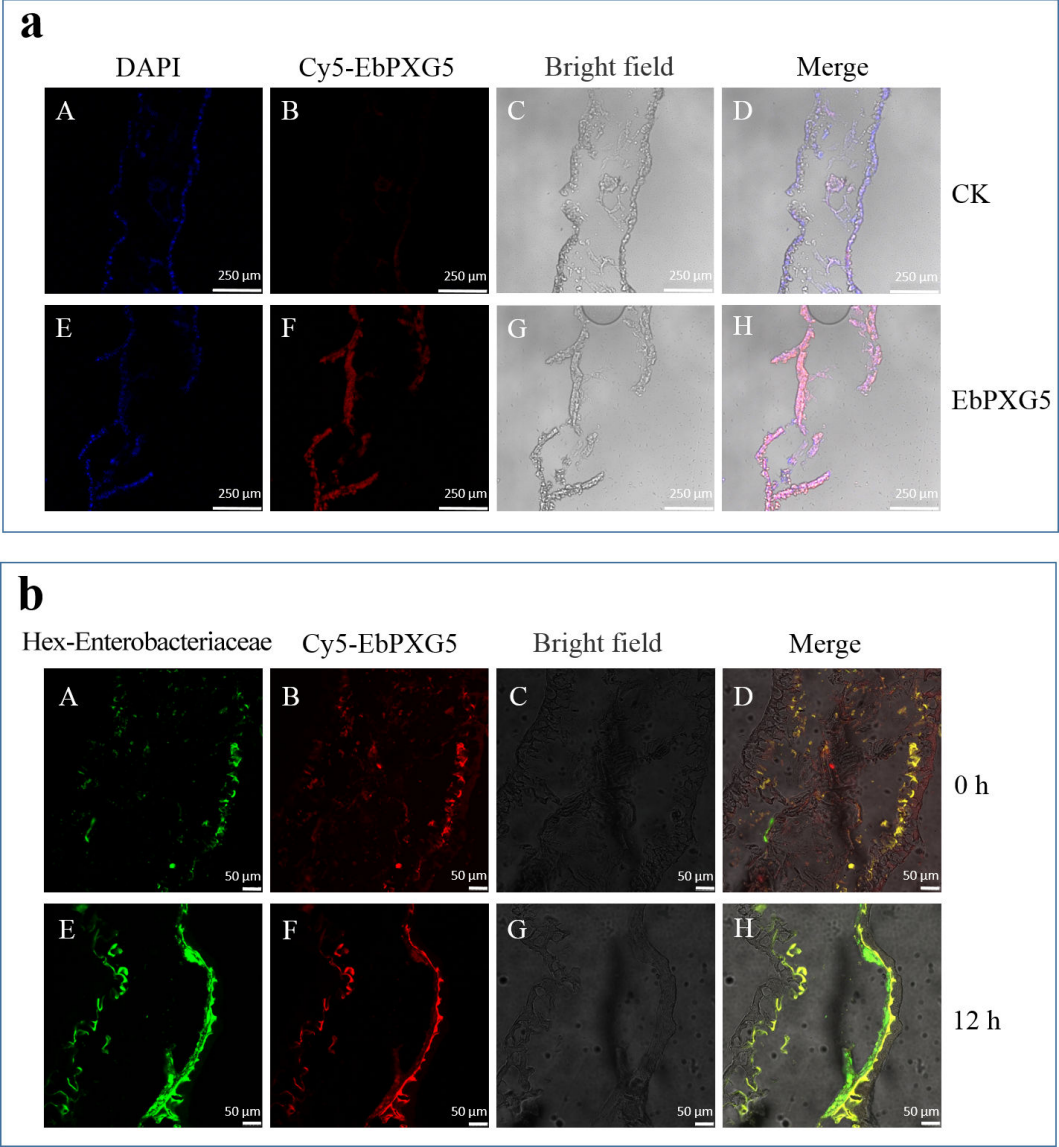
Colonization of EbPXG5 in the gut of *Plutella xylostella*. (a) *P. xylostella* reared under artificial diet. DAPI, DAPI staining of gut epithelial nuclei (**A and **E); Cy5-EbPXG5: the probe primers of EbPXG5 (Cy5-EbPXG5) labeled with red fluorescence Cy5 (B and F) and bright field (C and G); Merge: superposition of DAPI, Cy5-EbPXG5 markers, and bright field (D and H). (b) *P. xylostella* reared on radish seedling, A–D: 0 h starvation, E–H: 12 h starvation. Hex-Enterobacteriaceae: probe primers labeled with green fluorescence on Enterobacteriaceae (A and E); Cy5-EbPXG5: probe primers labeled with red fluorescence on EbPXG5 (B and F); bright field (C and G); Merge: superposition of bright field, hex-Enterobacteriaceae, and Cy5-EbPXG5 markers (D and H).

Fluorescence in situ hybridization (FISH) was used to assess whether EbPXG5 could establish in DBM. It was shown that this microbe colonized the gut of SL_SS_, distributed mainly along the gut epithelium, forming a stable biofilm (Fig. 6a). The distribution of EbPXG5 in the gut of DBM feeding on natural plants was analyzed by starving FZss individuals for 0 and 12 h. The results showed that EbPXG5 was mainly distributed along the FZss gut epithelium, which was consistent with SL_SS_. The comparison of starvation treatment for 0 and 12 h showed that there was a greater gut content at 0 h than at 12 h (basically empty). However, the colonization and distribution of EbPXG5 on the gut epithelium were not affected, indicating that EbPXG5 was prevalent in the DBM gut (Fig. 6b).

**Fig 7:**
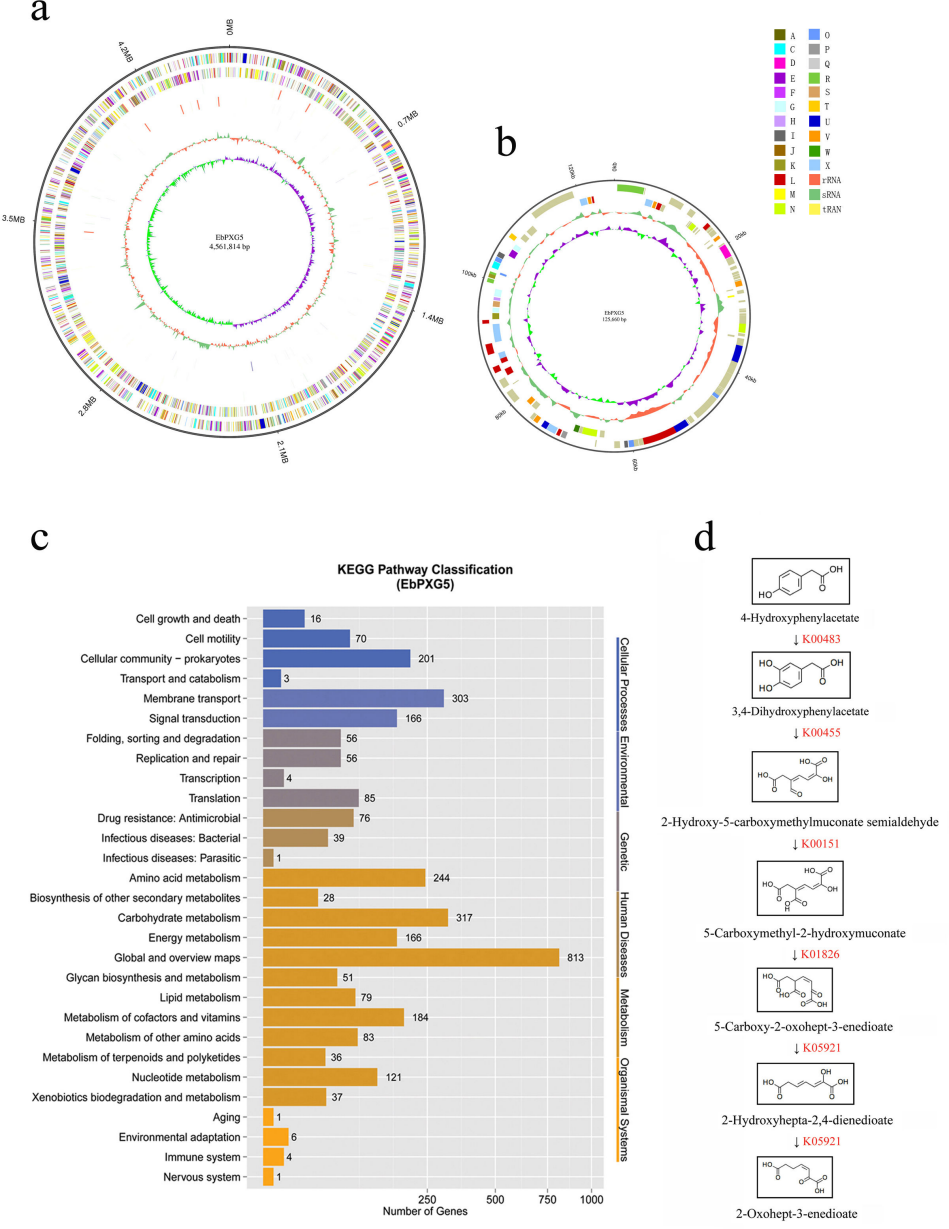
Genome analysis of *Enterobacter* sp. EbPXG5 (EbPXG5). (a) Circular representation of EbPXG5 genome. From the outside to the inside are (1) genome size, (2) forward strand gene, colored according to COG classification, (3) reverse strand gene, colored according to COG classification, (4) forward strand ncRNA, (5) reverse strand ncRNA, (6) repeat, (7) GC content, green indicates that the GC content in the region is higher than the average GC content of the entire genome, while red indicates that the GC content in the region is lower than the average GC content of the entire genome, (8) GC-SKEW (G-C/G + C), green indicates that the GC-SKEW value is <0, while purple indicates that the GC-SKEW value is >0. (b) Circular representations of EbPXG5 plasmid genome. From the outside to the inside are (1) genome size, (2) forward strand gene, colored according to COG classification, (3) reverse strand gene, colored according to COG classification, (4) GC content, (5) GC-SKEW (G-C/G + C). The colors in panels a and b of the COG classification represent (A) RNA processing and modification, (C) energy production and conversion, (D) cell cycle control, cell division, and chromosome partitioning, (E) amino acid transport and metabolism, (F) nucleotide transport and metabolism, (G) carbohydrate transport and metabolism, (H) coenzyme transport and metabolism, (I) lipid transport and metabolism, (J) translation, ribosomal structure, and biogenesis, (K) transcription, (L) replication, recombination, and repair, (M) cell wall/membrane/envelope biogenesis, (N) cell motility, (O) posttranslational modification, protein turnover, and chaperones, (P) inorganic ion transport and metabolism, (Q) secondary metabolites biosynthesis, transport, and catabolism, (R) general function prediction only, (S) function unknown, (T) signal transduction mechanisms, (U) Intracellular trafficking, secretion, and vesicular transport, (V) defense mechanisms, (W) extracellular structures, (X) mobilome: prophages and transposons. (c) KEGG pathway analysis of EbPXG5 genome; the digit on the column represents the number of genes. (d) The potential degradation pathway of aromatic compounds 4-hydroxyphenylacetate to 2-oxohept-3-enedioate, in which 3,4-dihydroxyphenylacetate 2,3-dioxygenase can catalyze the decomposition of benzene ring.

Genome sequencing was conducted to study the molecular mechanism of kaempferol degradation by EbPXG5. A total of 1,224 MB raw data, 802 MB effective data (clean data) were produced by Illumina (Table S2), and 1,885.5 MB Subreads data were obtained from PacBio platform (Table S3). The complete genome contained a 4.56 MB chromosome genome and a 0.126 MB plasmid genome (Fig. 7a and b; Table S4). By comparing the genome with the Nt database of NCBI, EbPXG5 was identified as *Enterobacter cloacae* encoding 4,360 genes (Table S5). COG analysis revealed that the EbPXG5 genome was rich in genes involved in metabolic function, and 95 genes participated in the secondary metabolism biosynthesis, transport, and catabolism pathway (Fig. S3).

KEGG analysis showed that the number of genes involved in metabolic function was the largest, accounting for 66.5% of the total genes (Fig. 7c), among which 37 genes were involved in xenobiotic biodegradation and metabolism, 36 genes participated in terpenoid and polyketide metabolism, 28 genes in the biosynthesis of other secondary metabolites, and 17 genes in the metabolism of aromatic compounds. These gene families mainly encoded for monooxygenase, dioxygenase, dehydrogenase, isomerase, reductase, amidohydrolase, acetyltransferase, aminotransferase, and methyltransferase, all involved in the degradation of aromatic compounds and other secondary metabolites (Table S6). In the degradation pathway of aromatic compounds, we found a potential metabolic pathway for the degradation of 4-hydroxyphenylacetate to 2-oxohept-3-enedioate, in which 3,4-dihydroxyphenylacetate 2,3-dioxygenase catalyzed the decomposition of benzene ring (Fig. 7d). According to the structure of the catalytic substrate of 3,4-dihydroxyphenylacetate, we speculated that the enzyme might possess the ability to catalyze the cleavage of the benzene ring in kaempferol and the related flavonoids; however, this still needs further validation. Furthermore, the genome analysis of EbPXG5 showed that urea carboxylase of the bacterium could convert cyanamide into allophanate (Table S6).

## DISCUSSION

DBM mainly feeds on cruciferous plants, which contain a high abundance of flavonoids (flavanol such as quercetin, kaempferol, and isorhamnetin) (15, 47). In this study, we found that these secondary metabolites (flavonoids generally but especially kaempferol) from radish seedlings reduced the weight of the fourth instar larvae of DBM and slowed larval development. However, gut bacteria (either the total gut bacteria or a single strain of EbPXG5) effectively alleviated the deleterious effects of plant secondary metabolites on DBM growth and development. Flavonoids are phenolic secondary metabolites widely distributed in the plant kingdom. Plants use these chemicals to protect against herbivores; the weight gain of silkworm (*Bombyx mori*) larvae decreases when fed on an artificial diet containing quercetin (48). Our study illustrates that gut bacteria can assist DBM to adapt to the host plant chemical defense. This represents a significant case of tri-partite co-evolution whereby an insect and its gut microorganisms (collectively the holobiont) adapt to counter host plant defense. This study identified several plant secondary metabolites from which the flavonoid kaempferol was selected for further study, reflecting the fact that flavonoids are important high-abundance secondary metabolites in cruciferous vegetables (15, 16). Furthermore, previous studies have shown that another lepidopteran (“caterpillar”) insect herbivore, *Pieris brassicae,* which, like DBM, specializes in brassica plants, but, unlike DBM, exhibits inhibited growth and feeding in the presence of kaempferol (49).

Our previous studies have shown that Enterobacteriaceae dominate the DBM gut ecology and participate in the degradation of cellulose, xylan, pectin, and phenol (27). In the present study, the abundance of Enterobacteriaceae in the gut of DBM was upregulated by kaempferol, and EbPXG5 had the ability to degrade kaempferol *in vivo* and *in vitro*. Although we did not find a complete metabolic pathway that could directly degrade kaempferol in EbPXG5, genome analysis found many enzymes involved in the degradation of terpenoids and polyketones, especially those that may cleave the benzene ring in kaempferol and the related flavonoids. In addition, the genome analysis still showed the ability of EbPXG5 to convert cyanamide into allophanate. Cyanamide is a toxic secondary metabolite in many plants, which can even be used as propesticide against phytophagous insects (50, 51). This suggests that EbPXG5 may play a role in helping host insects adapt to plant nitrile compounds. Our results combined with previous studies further confirm that EbPXG5 has a broader spectrum of functions in the detoxification of plant secondary metabolites. Cheng et al. report that the invasive pest *Dendroctonus valens* endangers the host plant *Pinus tabuliformis* by inducing the host to produce a secondary metabolite naringen to defend against *D. valens*, while the presence of the symbiotic Gram-negative bacteria *Novosphingobium* sp. can help *D. valens* degrade naringin and improve its survival rate (52). Colorado potato beetle (*Leptinotarsa decemlineata*) larval microbial symbionts can suppress the anti-herbivore defenses in tomato (*Solanum lycopersicum*), favorizing larval growth (53). Simons et al. report that even human gut microorganisms can degrade flavonoids including apigenin, genistein, naringin, and kaempferol (54). It is suggested that herbivorous animals’ gut bacteria may be universal in the degradation of plant secondary metabolites. Enterobacteriaceae (such as EbPXG5) is a widespread family in the gut of DBM. The gut of DBM is well-suited to being colonized by such bacteria. In this experiment, the bacterial population established in the gut of DBM after feeding (DBMT.CK) is shown not to be consistent with the identity of the bacteria fed (DBMT), regardless of the population diversity or specific bacterial abundance. This shows that the DBM gut had a certain selective function for bacterial colonization. It could receive foreign microorganisms, but not unconditionally through a process of reshaping microbial diversity in its gut. FISH analysis revealed that EbPXG5 colonized the gut and distributed along its epithelium, forming a stable biofilm to protect the host insect, which indicated that the gut of DBM is adapted to maximize the adaptive advantage provided by this microbial flora. We used the Enterobacteriaceae probe instead of the universal probe because the Enterobacteriaceae is abundant in the gut of the DBM [presence in the DBM larvae gut as high as 85.7% (27)], and it is more convenient to see that EbPXG5 is not the only *Enterobacter* in the gut. While FISH experiments are potentially open to errors and interference caused by non-specific fluorescence, such as autofluorescence of insect tissue, however, when interpreted in comparison with the sterile DBM control, it still provides evidence of adaptation.

*Enterobacter* is common in functional flora of insect gut, such as *Bactrocera dorsalis* (55, 56), *S. litura* (34), *Spodoptera littoralis* (57), and *Gryllotalpa krishnani* (58). Enterobacteriaceae is one of the most widespread families across 30 lepidopteran insects, while the *Enterobacter* is one of the most common genera (59). This symbiosis between Lepidoptera and *Enterobacter* suggests that they may form a mutually beneficial cooperative relationship in their long-term co-evolution process. The gut microorganisms of foliar-feeding insects mainly come from the soil via the host plant (60). *Enterobacter* is widely distributed in soil and plant leaves. Our previous study shows that the establishment of gut microbes of the DBM is closely related to diet (61). In addition, taking *Enterobacter* as a model, it was found that the gut microbes of DBM derived from diet can enter the ovary, reach the egg, and then transmit vertically to the next generation as well as transmitting horizontally (62). Wild populations of DBM are readily able to obtain such microorganisms from their host plants and benefit from a protective barrier and detoxification, promoting development. The number of genes encoded by the gut microbiome of DBM is equivalent to that encoded by its own genome (27). This biome-mediated adaptation to the host plant defenses is an elegant method to quickly adapt to selection pressure without changing the host’s own genes. Our work shows a particular case of the phenomenon that the gut microorganisms in insects use to deal with plant secondary metabolites.

While this study shows that EbPXG5 can utilize kaempferol and reduce its toxicity, there are still some shortcomings. Since the metabolic pathway and functional genes involved in degrading the toxic plant compound were identified in EbPXG5 based on genomic analysis, confirmatory studies are necessary to reintroduce a mutant strain (such as deleting the key metabolic gene) into the host to provide conclusive evidence of this universality. Nevertheless, removing kaempferol is complex and can include transformation to other secondary metabolites, such as quercetin; ring opening cracking; addition of groups, such as the formation of sulfate, methyl, and glucuronic acid conjugates, etc. (63, 64). Although we have a genome and derive potential ring-opening cleavage pathways, these pathways and enzymes can only indicate that they possess the potential to degrade aromatic compounds. Several other genes in the genome or other mechanisms that have the potential to degrade xenobiotics exist. We currently do not know exactly which genes and pathways could be involved in the degradation process of kaempferol, and the molecular mechanism of kaempferol degradation by EbPXG5 is in fact not clear yet. The presence of many genes, however, related to the degradation potential of xenobiotics in this bacterium, is consistent with the experimental results. Our results are supportive of elucidating the co-evolution of gut bacteria and insect hosts. The review focused on the Lepidoptera microbiome also summarizes many examples of gut microbiota mediating host insect detoxification of plant toxins and xenobiotics (65), which is a strong support for our conclusion. Second, since the degradation of kaempferol might not be specific to EbPXG5, we did not exclude other bacteria that may degrade kaempferol in this study. As the metabolic potential of microorganisms is huge, it is unlikely that EbPXG5 is the only bacterium in the gut of DBM, which can degrade kaempferol or other secondary metabolites. EbPXG5 was selected as the target bacterium for this study because we found that *E. cloacae* was the most abundant species in the gut of DBM larvae, accounting for 39.2% of the total abundance by metagenomics analysis of the gut microorganisms, and the metagenomics analysis suggested that this species might have the metabolic potential to cleave aromatic compounds, which might play an important role in the defense of plant secondary metabolites (27). EbPXG5 was identified as *E. cloacae* after genome sequencing and comparison with the NCBI database, so this species and DBM were selected as a model to study its co-evolution, to clarify that the gut bacteria of DBM really play a role in assisting the host to adapt to the plant secondary metabolites and clarify the significance of this co-evolution. In addition, kaempferol may also not be the only secondary metabolite that has inhibitory effects on insects. Other compounds, such as quercetin, which has a very similar structure to kaempferol, have been shown to have inhibitory effects on the growth and development of insects (66, 67). To expand on this model, other compounds should be included in future studies. In spite of this, this work does show a very interesting effect even if we do not know if the bacteria or kaempferol we tested here is unique or not. Our work represents an important step forward in understanding by showing the effects of a particularly dominant bacterium and plant secondary metabolites. Disruption of this mechanism in pest species by targeting the gut microbiome offers potential for novel plant protection strategies, while strategic enhancement of the microbiome may offer scope for the protection of beneficial insects such as pollinators or natural enemies.

## Data Availability

The raw sequences of gut microbiota of *P. xylostella* were deposited to the NCBI Sequence Read Archive (SRA) database with the sample accession numbers SAMN11269626–SAMN11269634. The raw sequences of EbPXG5 genome were deposited to the NCBI SRA database with the BioProject accession number PRJNA791514.

## SUPPLEMENTAL MATERIAL

Supplemental material msystems.00826-23-s0001.docx Fig. S1 to S3; Tables S2 to S5.

Table S1 msystems.00826-23-s0002.xlsx Composition analysis of secondary metabolites in radish seedling by LC-MS.

Table S6 msystems.00826-23-s0003.xlsx Genomic analysis of the genes involved in the biodegradation of plant secondary metabolites by Enterobacter sp. EbPXG5.

ASM does not own the copyrights to Supplemental Material that may be linked to, or accessed through, an article. The authors have granted ASM a non-exclusive, world-wide license to publish the Supplemental Material files. Please contact the corresponding author directly for reuse.
