# Supplementary material for: Gut bacteria mediated adaptation of diamondback moth, Plutella xylostella, to secondary metabolites of host plants
Source: mSystems. 2023 Nov 1;8(6):e00826-23. doi: 10.1128/msystems.00826-23 (PMC10734469; doi:10.1128/msystems.00826-23)
Supplement: Supplemental material — Fig. S1 to S3; Tables S2 to S5. [file msystems.00826-23-s0001.docx]

**Supplementary Information**

**Gut bacteria mediated adaptation of diamondback moth, *Plutella xylostella*, to secondary metabolites of host plants**

Xiaofeng Xia^1,2,3,4#,*^, Qian Wang^1,2,3,4#^, Geoff M. Gurr^1,2,3,5^, Liette Vasseur^1,2,3,6^, Shuncai Han^1,2,3,4^, Minsheng You^1,2,3,4*^

^1^State Key Laboratory of Ecological Pest Control for Fujian and Taiwan Crops, Institute of Applied Ecology, Fujian Agriculture and Forestry University, Fuzhou 350002, China;

^2^International Joint Research Laboratory of Ecological Pest Control, Ministry of Education, Fujian Agriculture and Forestry University, Fuzhou 350002, China;

^3^Key Laboratory of Integrated Pest Management for Fujian-Taiwan Crops, Ministry of Agriculture, Fuzhou 350002, China;

^4^Fujian-Taiwan Joint Innovation Centre for Ecological Control of Crop Pests, Fujian Agriculture and Forestry University, Fuzhou 350002, China;

^5^Graham Centre, Charles Sturt University, Orange, New South Wales 2800, Australia;

^6^Department of Biological Sciences, Brock University, 1812 Sir Isaac Brock Way, St. Catharines, Ontario, Canada L2S 3A1.

**Running Head: DBM gut microbiota for plants secondary metabolites**

^*^Correspondence to X.X. (Email: xiaofengxia@fafu.edu.cn) and M.Y. (Email: msyou@fafu.edu.cn).

^#^These authors contributed equally to this work.

**This file includes:**

Supplementary Figures 1-3

Supplementary Tables 2-5


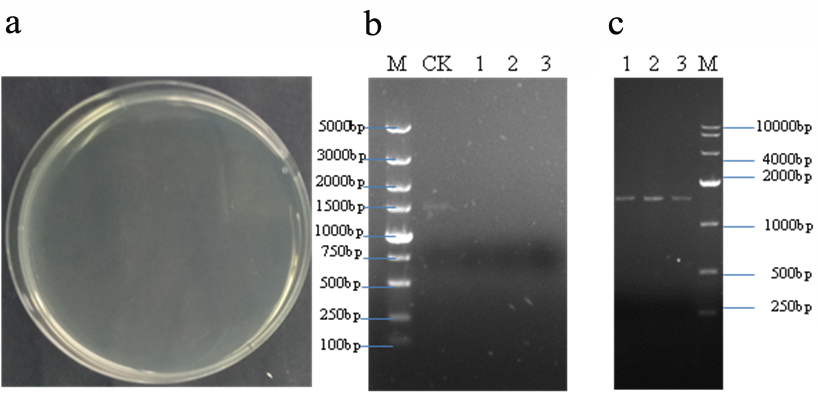


**Figure S1 Detection of gut bacteria in *Plutella xylostella*.** a. the sterile *P. xylostella* gut contents cultured on LB medium. b. M: Marker, CK: 16S rDNA detection of the ddH_2_O after disinfection of body surface of *P. xylostella*, 1-3: 16S rDNA detection of the *P. xylostella* gut contents under aseptic feeding. c. M: Marker, 1-3: 16S rDNA detection of the *P. xylostella* gut contents under non-aseptic feeding.


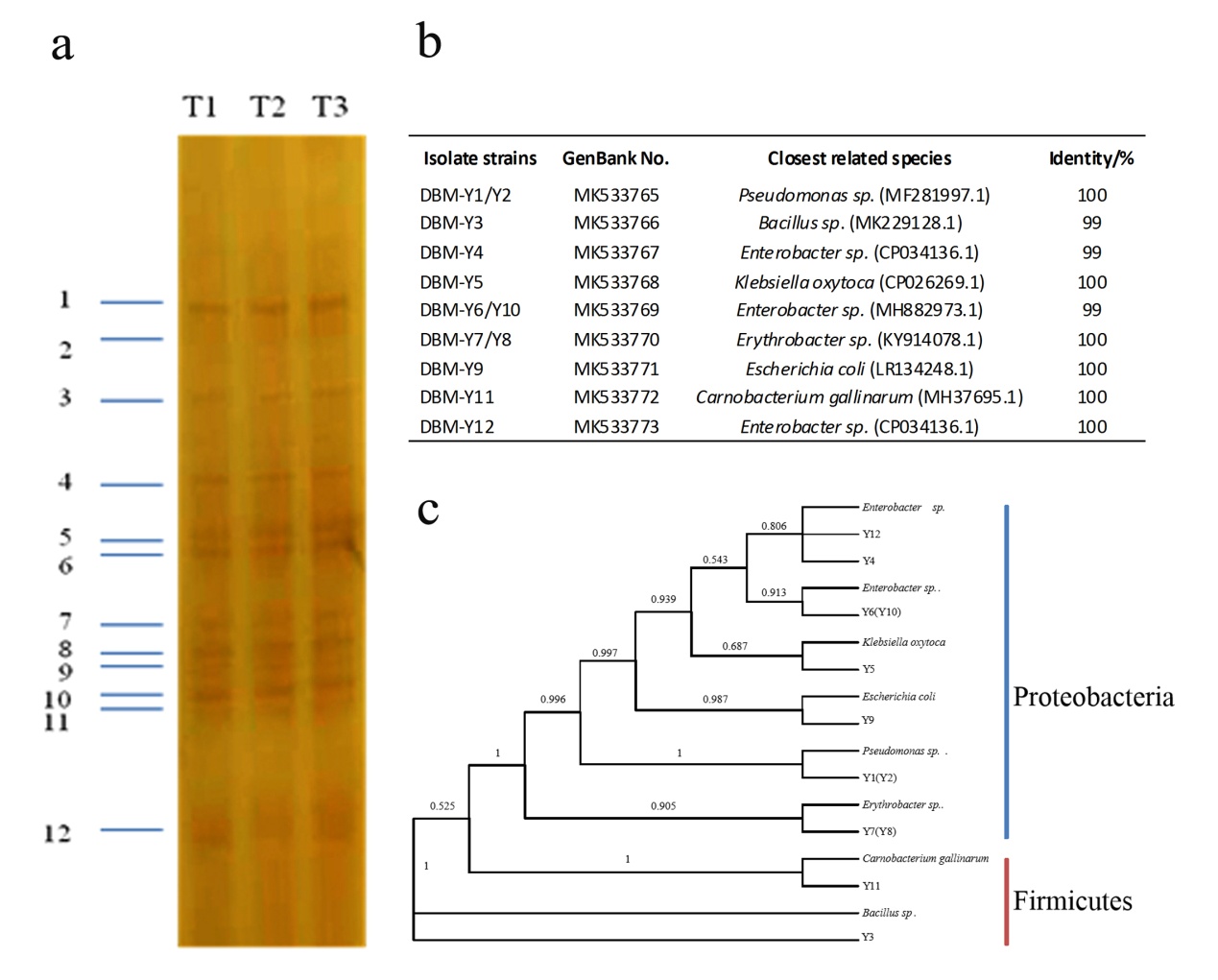


**Figure S2 PCR-DGGE analysis of the *Plutella xylostella* gut bacteria recovered from Luria-Bertani (LB) enrichment culture**. a: PCR-DGGE fingers of the *P. xylostella* gut bacteria; b: Identification of the gut bacteria based on the V3 region of 16S rDNA recovered from the bands in DGGE fingerprints; c: Phylogenetic analysis of the *P. xylostella* gut bacteria recovered from DGGE.


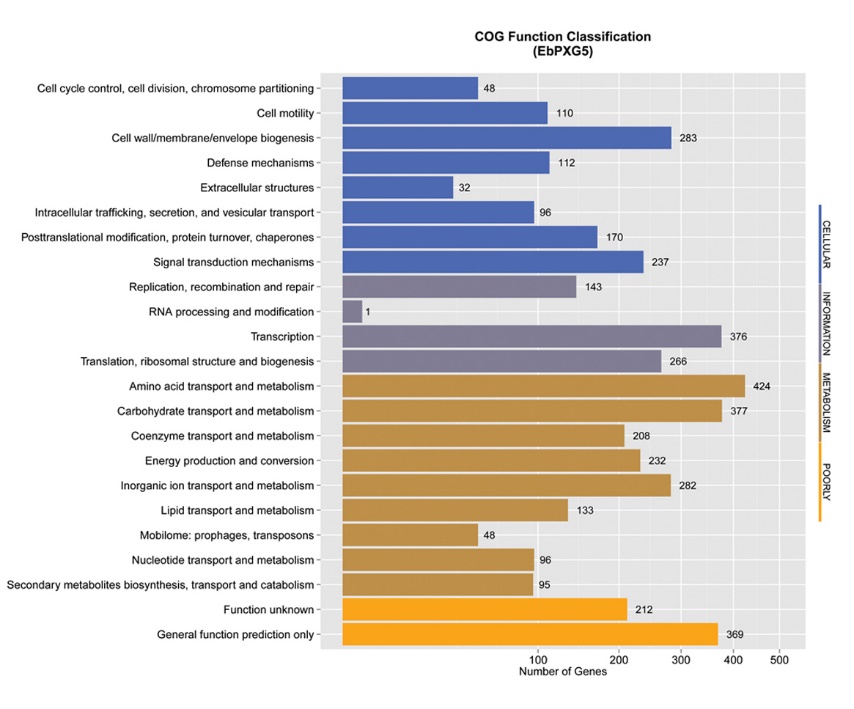


**Figure S3 COG functional analysis of EbPXG5 genome.** The digits on the column represent the number of genes.

**Table S2 Data of the Illumina sequencing of EbPXG5**

| Sample  ID | Insert  Size(bp) | | Raw  Data(Mb) | Adapter  (%) | | | Duplication of the same reads  (%) | | Total  Reads | Filtered  Reads (%) | | Clean Data(Mb) |
| --- | --- | --- | --- | --- | --- | --- | --- | --- | --- | --- | --- | --- |
| EbPXG5 | | 270 | 1224 | | 0.13 | 6.06 | | 8,164,322 | | | 21.40 | 802 |

**Table S3 Data of the PacBio sequencing of EbPXG5**

| Sample  ID | Subreads total Bases (Mb) | | Subreads Number | Subreads Mean length (bp) | | N50  length (bp) | | N90  length (bp) | Max size of Subreads (bp) | |
| --- | --- | --- | --- | --- | --- | --- | --- | --- | --- | --- |
| EbPXG5 | | 1885.5 | 309,426 | 6,093 | 7,385 | | 3,797 | | | 52,271 |

**Table S4 Assembly statistics of EbPXG5**

| Sample  ID | ID Name | | Sequence Topology | | Sequence Number | | Total  Length (bp) | | | GC content  (%) |  |
| --- | --- | --- | --- | --- | --- | --- | --- | --- | --- | --- | --- |
| EbPXG5 | | Chromosome1  Plasmid1  All | | circular  circular | | 1  1  2 | | 4,561,814  125,680  4,687,474 | 56.07  52.91  55.98 | | |

**Table S5 Information of the genes in EbPXG5**

| Sample  ID | Genome Size | | Total  Gene Number | | | Total Length of genes | Average  Length of genes (bp) | | | GC content of genes  (%) |  |
| --- | --- | --- | --- | --- | --- | --- | --- | --- | --- | --- | --- |
| EbPXG5 | | 4,687,474 | | 4,360 | 4,132,170 | | | 947.75 | 57.27 | | |
